# Inhibiting ADORA1 enhances glioma apoptosis and increases its sensitivity to anti-PD1 therapy

**DOI:** 10.3389/fonc.2025.1545780

**Published:** 2025-05-01

**Authors:** Hong-jiang Li, Zhi-yun Yu, Hua-ping Gao, Yi-ran Xu, Xue-yuan Li, Wei Jiang, Di Chen, Dong-ming Yan, Chao Yang, Xian-zhi Liu

**Affiliations:** ^1^ Department of Neurosurgery, The First Affiliated Hospital of Zhengzhou University, Zhengzhou, China; ^2^ Henan Key Laboratory of Child Brain Injury and Henan Clinical Research Center for Child Neurological Disorders, Institute of Neuroscience, Third Affiliated Hospital of Zhengzhou University, Zhengzhou, China; ^3^ The Application Center for Precision Medicine, Academy of Medical Science, Zhengzhou, China

**Keywords:** glioma, ADORA1, apoptosis, PD1, kininogen-1

## Abstract

**Introduction:**

Glioma, the primary cancerous tumor of the central nervous system in adults, has a poor outlook. Immune checkpoint blockade therapy has exhibited notable efficacy against various cancer types. Prior research has suggested that the adenosine A1 receptor (ADORA1) facilitates the proliferation of tumors in cancer. Nevertheless, the precise impact of ADORA1 on glioma progression and its influence on anti-programmed death receptor 1 (PD1) therapy, along with the underlying regulatory mechanisms, remain to be fully elucidated.

**Methods:**

Bioinformatics was used to explore the correlation between ADORA1 expression and glioma prognosis. The effects of ADORA1 on glioma and anti-PD1 therapy were investigated in both laboratory settings and living organisms.

**Results:**

The results revealed a significant increase in ADORA1 expression in glioma, which was correlated with poor prognosis. Furthermore, ADORA1 inhibition facilitated glioma apoptosis by augmenting kininogen-1 (KNG1). ADORA1 inhibition enhanced T cell recruitment and increased glioma susceptibility to anti-PD1 therapy.

**Dicussion:**

Our findings indicate that inhibiting ADORA1 can induce apoptosis in glioma cells and increase their sensitivity to anti-PD1 therapy. ADORA1 may serve as a prognostic marker for glioma and a potential target to enhance the effectiveness of anti-PD-1 therapy.

## 1 Introduction

Gliomas are the most common primary brain tumors in adults, with patients with glioblastoma (WHO IV) experiencing median overall survival remains approximately 15 months despite standard treatments of surgery, radiotherapy, and chemotherapy (1). Consequently, there exists an imperative need to investigate the fundamental molecular mechanisms underlying the progression of glioma malignancy, with the aim of devising more effective therapeutic interventions.

Novel immune and targeted therapy strategies have emerged, offering promising avenues for advancing GBM treatment (2). Immune checkpoint blockade therapy, which targets the programmed death receptor 1 (PD-1) and its ligand (PD-L1) pathway, has demonstrated considerable efficacy in the treatment of various malignancies (3, 4). Nevertheless, GBM is largely refractory to antibodies against programmed cell death 1 (anti-PD-1) therapy (5). The existing response rates continue to be unsatisfactory because of resistance to anti-PD1/PD-L1 treatment and the absence of suitable biomarkers for categorizing patients (2, 6). Hence, it is crucial to identify accurate and efficient targets to overcome resistance.

**Figure 1:**
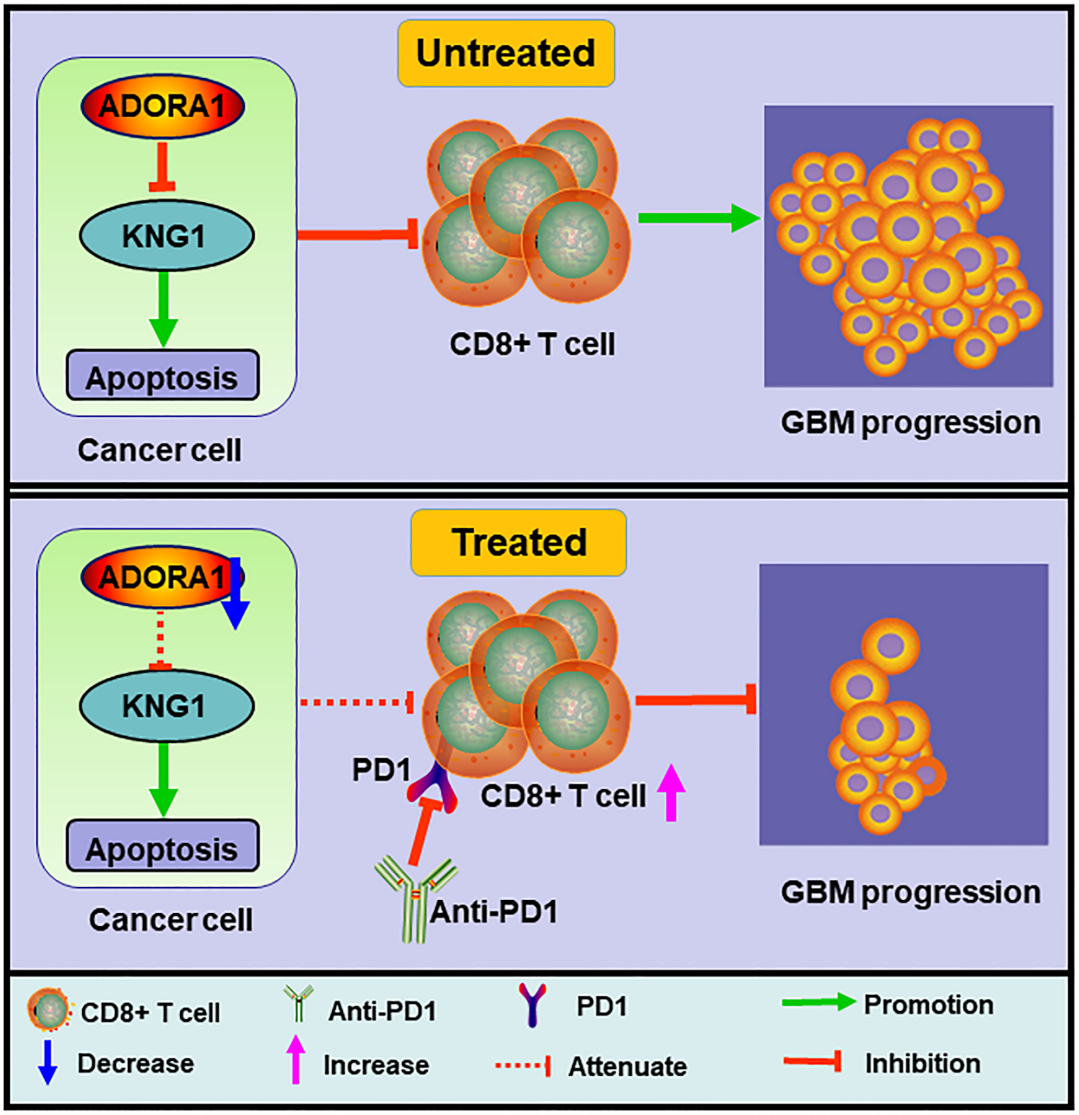
Schematic diagram of the mechanism. ADORA1 inhibition promoted glioma apoptosis, enhanced CD8+T cell expression, and sensitized glioma to anti-PD1 therapy.

The adenosine A1 receptor (ADORA1), which functions as a receptor for adenosine, has been documented to exhibit pro-tumor growth effects in breast carcinoma, renal cancer, and hepatocellular carcinoma (7–9). Previous studies have indicated high ADORA1 expression in high-grade gliomas; however, its specific role in glioma progression and the efficacy of glioma immunity therapy remain unclear. The objective of this study was to examine the influence of ADORA1 on the progression of glioma and its possible consequences for anti-PD1 treatment (**Figure 1**).

## 2 Materials and methods

### 2.1 Clinical samples and animals

Histologically diagnosed glioblastoma (WHO IV) samples (n=3) were acquired from the First Affiliated Hospital of Zhengzhou University, conducted in accordance with the Declaration of Helsinki and written informed consent was provided to all patients. 4-week-old C57BL/6N mouse used in this study were purchased from GemPharmatech Co. Ltd. The study was approved by the Ethics Committee of the First Affiliated Hospital of Zhengzhou University (approved number: 2019-KY-176).

### 2.2 Cell culture

The GBM-Z1 glioma cell line was derived from surgically removed human glioma tissues that were histologically diagnosed as GBM. The U87 and GL261 glioma cell lines were obtained from the Cell Library of the Chinese Academy of Sciences in Shanghai, China. GBM-Z1 cells were cultured in DMEM/F12 medium supplemented with 10% fetal bovine serum (FBS; Thermo Fisher Scientific, USA). GL261 cells were cultured in DMEM medium supplemented with 10% FBS (Thermo Fisher Scientific, USA).

In GBM-Z1 and U87 cell lines, the cDNA sequences of human ADORA1 (NCBI Gene ID: 134) and KNG1 (Gene ID: 3827) were cloned into vectors to facilitate overexpression. Similarly, in GL261 cells, the cDNA sequences of ADORA1 (NCBI Gene ID: 11539) and KNG1 (Gene ID: 16644) were cloned into vectors for the purpose of overexpression.

### 2.3 Bioinformatics analysis

We investigated the expression or clinical data of ADORA1 in glioma using data from The Cancer Genome Atlas (TCGA) (https://www.cancer.gov/), Chinese Glioma Genome Atlas (CGGA) (http://www.cgga.org.cn/), and Genotype-Tissue Expression (GTEx) databases (https://www.gtexportal.org/). The CGGA provides mRNA expression (CGGA325) and clinical data. Patients with glioma were categorized into high or low risk groups based on the median risk score, then the R package “survival” is used to analyze the survival curve (10). The prognostic value of the ADORA1-based classifier was assessed via receiver operating characteristic (ROC) curve analysis. The R programming language includes a software package named “timeROC” (11). The analysis considered timeframes of 1, 3, and 5 years for this objective.

### 2.4 Immunofluorescence assay

Mouse were euthanized with isoflurane and then injected with 0.9% normal saline plus 4% paraformaldehyde through the heart. Xenograft tissue samples were fixed overnight and immersed in 30% sucrose and 0.1 M phosphate-buffered saline (PBS) solution for 48 h. The specimens were frozen at –80°C with optimal cutting temperature compound, and 8-μm coronal sections were prepared using a cryostat microtome (CM 3050S Leica, Germany). The sections were treated with 0.5% Triton X-100 (Solarbio, Beijing, China) in PBS for 30 min. Anti-CD4 antibody (1:200, ab288724, Abcam), anti-CD31 antibody (1:200, 66065-2-Ig, Proteintech) and anti-CD8 antibody (1:200, ab217344, Abcam), were applied using a blocking solution of 3% horse or fetal bovine serum and 0.3% Triton X-100 and incubated overnight at 4°C. The secondary antibody, Alexa Fluor 555 (1:1000, Thermo Fisher Scientific), was used. The sections were washed three times with PBS and mounted in a medium containing 4′,6-diamidino-2-phenylindole (DAPI) to identify the nucleus.

### 2.5 Immunohistochemistry and HE staining

Animals were sacrificed, and brain tissue samples were fixed and processed into 15-μm sections. The sections underwent immunohistochemical (IHC) staining, including deparaffinization, rehydration, antigen retrieval, endogenous peroxidase quenching, and blocking. The sections were incubated overnight at 4°C with primary antibodies against ADORA1 (1:200, ab3460, Abcam) and SOX2 (1:200, MA1-014, Thermo Fisher). Following incubation, the sections were treated with a secondary antibody for 50 mins and visualized using DAB chromogenic solution. The hematoxylin and eosin (HE) staining method was used to select slides with the largest tumor area, and images were obtained using a light microscope.

### 2.6 Gene knockdown and overexpression

Obio Technology Co., Ltd. (Shanghai, China) constructed lentiviruses for ADORA1 overexpression and knockdown, which were transfected into glioma cells.

### 2.7 Western blotting and immunoprecipitation

The manufacturer’s guidelines were followed when conducting the western blot and IP experiments. To conduct western blot analysis, all the tissue samples were added to two hundred microliters of RIPA buffer and shaken for 1 min after ultrasound (on for 2 s, off for 3 s). the cell samples were treated with RIPA buffer and lysed for 30 min. Subsequently, the tissue and cell samples were centrifuged at 12,000 ×*g* for 10 min at 4°C. Post-separation, the supernatant was electro-blotted and incubated with antibodies targeting ADORA1 (1:1000, ab3460, Abcam), kininogen-1 (KNG1) (1:1000, ab124737, Abcam), and GAPDH (1:1000, ab8245, Abcam). Following incubation, the cells were treated with horseradish peroxidase-conjugated secondary antibodies. An ultrasensitive enhanced chemiluminescence detection kit was used. Immunoreactive bands were quantified using ImageJ software.

In the IP assay, cells were homogenized in Co-IP buffer and centrifuged at 12,000 ×*g* for 10 min at 4°C. The supernatant was incubated with antibodies for 2 h, followed by the addition of Protein G beads, which were left overnight at 4°C. Subsequently, the beads were subjected to SDS-PAGE and were subsequently transferred to nitrocellulose membranes. The collected data were analyzed using ImageJ software.

### 2.8 Colony formation assay

GBM-Z1 cells were transfected with LV-control, LV-ADORA1, and LV-shADORA1. The cells were treated with 4% PFA and then stained with 0.1% crystal violet. A digital camera was used to capture images of the colony.

### 2.9 Transwell migration and invasion assays

GBM-Z1 cells were introduced into the upper compartment of a Transwell chamber (Corning, USA) for migration and invasion assays, with Matrigel mix (Corning, USA) added as required. GBM-Z1 (2×10^4^ cells/well) were suspended in low-serum (2% FBS) medium and seeded in the upper chamber. The lower chamber was filled with DMEM supplemented with 10% FBS. After 48 h of incubation, the cells that traversed the membrane were fixed with 4% PFA and stained with 0.1% crystal violet. Images were obtained using an inverted microscope.

### 2.10 Intracranial glioma model

To construct intracranial glioma models, mouse were anesthetized with isoflurane prior surgery. Mice of comparable weight and age were randomly assigned to three distinct experimental groups. 5 × 10^5^ GL261 cells were injected into the striatum of mice at 0.25 μl/min using a stereotactic frame and a microinfusion syringe pump. The tumor development was assessed using bioluminescence imaging 3 weeks after implantation.

### 2.11 Statistical analysis

GraphPad Prism 6 was used for statistical analysis. A minimum of three replicates were conducted for each experiment, and the results are presented as the average ± standard deviation. Statistical significance was assessed using both the Student’s *t* test and one-way ANOVA. A *P* value < 0.05 was considered statistically significant.

## 3 Results

### 3.1 *ADORA1* is associated with prognosis of glioma

**Figure 2:**
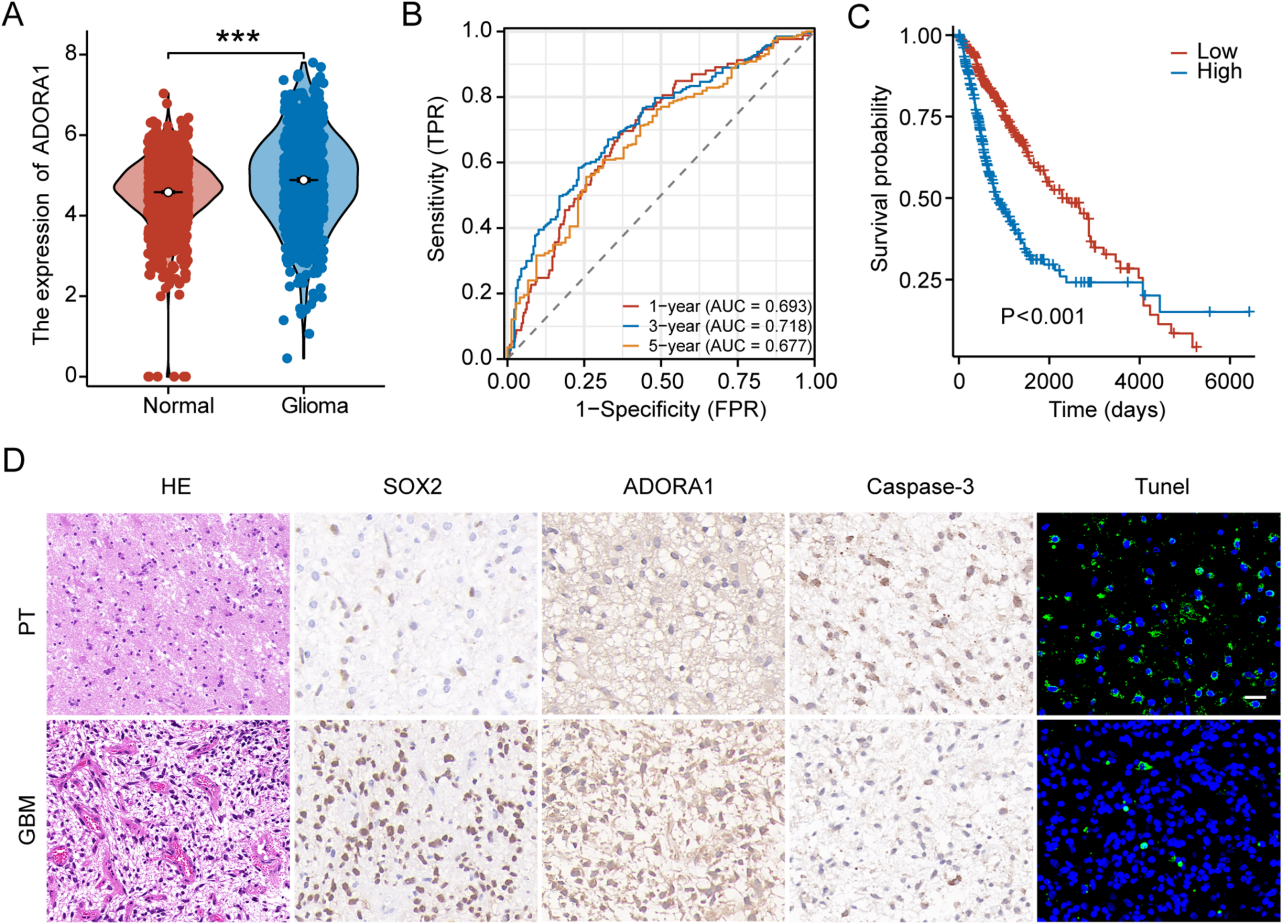
ADORA1 is highly expressed and associated with prognosis in glioma. **(A)** The gene expression levels of ADORA1 between normal and GBM tissues were compared based on TCGA database. **(B)** Receiver operating characteristic curve analysis verified the prognostic performance of the risk score based on ADORA1 in TCGA database. **(C)** Correlation between ADORA1 and survival based on TCGA database. **(D)** Immunohistochemistry and HE staining in human GBM and paratumor (PT) (scale bars: 20 μm). ***p<0.001.

TCGA and GTEx databases, which have more than 1800 samples, were selected and showed that the gene expression of *ADORA1* was significantly higher in gliomas than in normal tissues (**Figure 2A**). Time-dependent ROC analysis using TCGA was performed to assess the prognostic significance of ADORA1-derived risk scores. In TCGA database, the risk scores achieved Area Under the Curve (AUC) of 0.693, 0.718, and 0.677 for predicting the 1-, 3-, and 5-year overall survival, respectively (**Figure 2B**). Survival analysis of patients with glioma from TCGA database stratified into high- and low-risk groups revealed that those with elevated ADORA1 expression had a worse prognosis (**Figure 2C**). SOX2, and caspase3 were selected as principal biomarkers due to their well-documented roles in tumor stem cell maintenance, apoptotic processes, and microenvironment regulation, respectively. In GBM samples derived from three patients, HE staining revealed a remarkably dense population of cells and excessive growth of vascular endothelial cells, along with abnormal changes in the nuclei, such as increased darkness and atypical cell division (**Figure 2D**). IHC staining revealed a pronounced presence of SOX2 in human GBM tissues, whereas the expression of ADORA1 was notably higher in GBM tissues compared to paratumoral tissues (**Figure 2D**).

### 3.2 Assessment of the clinical relevance of ADORA1

**Figure 3:**
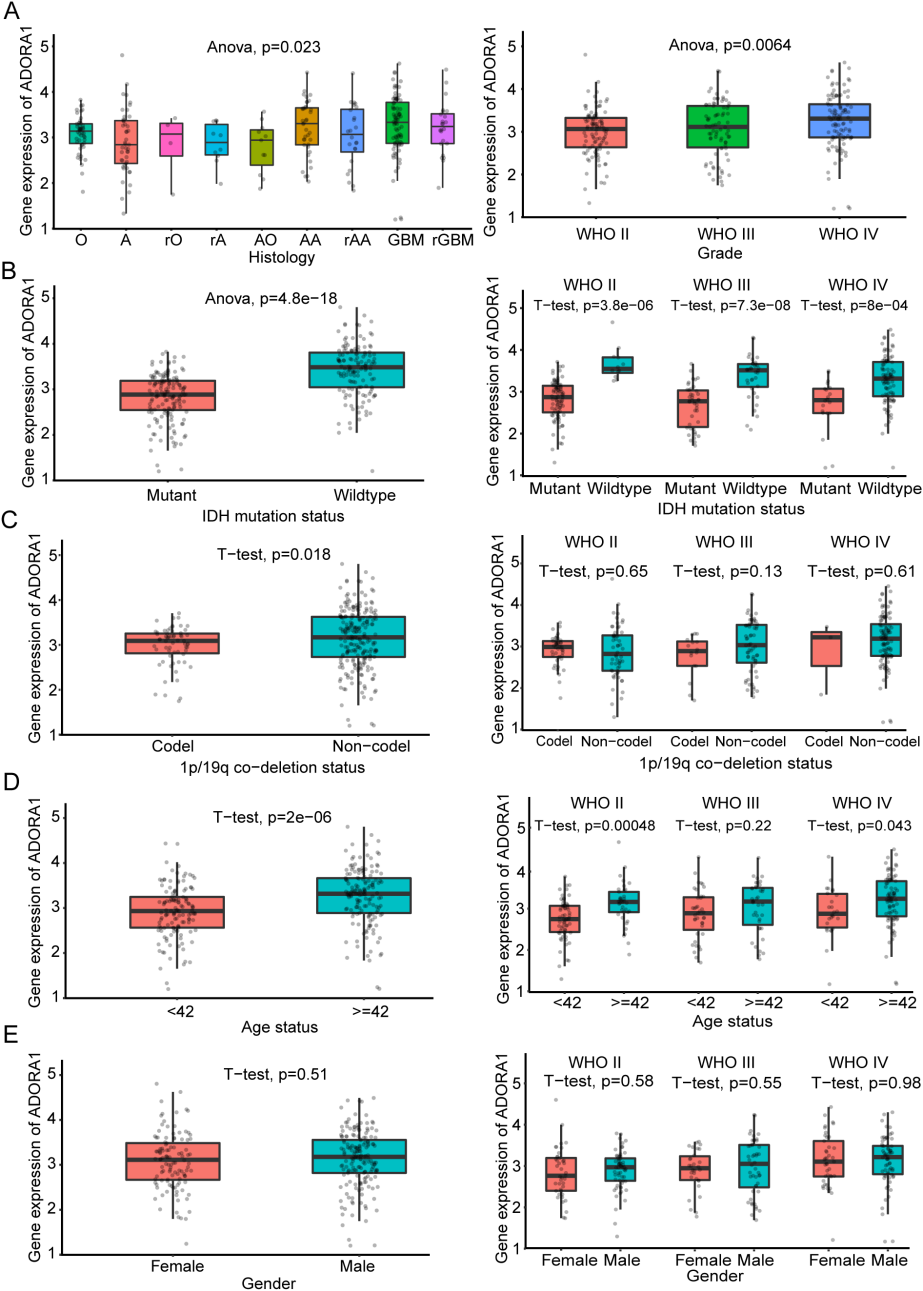
Clinical significance of ADORA1 according to the CGGA325 database. The correlation of ADORA1 with different types of gliomas and **(A)** World Health Organization grade (A, strocytoma; rA, recurrence strocytoma; O, oligodendroglioma; rO, recurrence oligodendroglioma; AO, Anaplastic oligodendro; AA, anaplastic astrocytoma; rAA, recurrence anaplastic astrocytoma; GBM, primary glioblastoma; sGBM, secondary glioblastoma; rGBM, recurrence of GBM) **(B)** IDH status, **(C)** 1p/19q co-deletion status, **(D)** age, and **(E)** sex are shown in the CGGA325 database.

We analyzed *ADORA1* expression levels using the CGGA database to investigate the impact of *ADORA1* expression on glioma progression. According to these findings, GBM exhibited a higher *ADORA1* expression level than other types of glioma, and there was a positive correlation between *ADORA1* and the grade of glioma (**Figure 3A**). IDH mutation and 1p/19q co-deletion are key markers associated with favorable prognosis in glioma. In gliomas lacking IDH1/2 mutations and 1p/19q co-deletion, elevated *ADORA1* expression was observed, suggesting a negative correlation with prognosis (**Figures 3B, C**). The clinical data compiled within the CGGA database indicates that the mean age of participants was 42 years. Moreover, individuals aged > 42 years exhibited increased *ADORA1* expression (**Figure 3D**). Additionally, *ADORA1* expression was not significantly correlated with sex (**Figure 3E**). These findings highlight ADORA1 as a potential marker linked to aggressive glioma biology and poorer outcomes in specific molecular.

### 3.3 *In vitro*, ADORA1 facilitates the growth, migration, and infiltration of glioma

**Figure 4:**
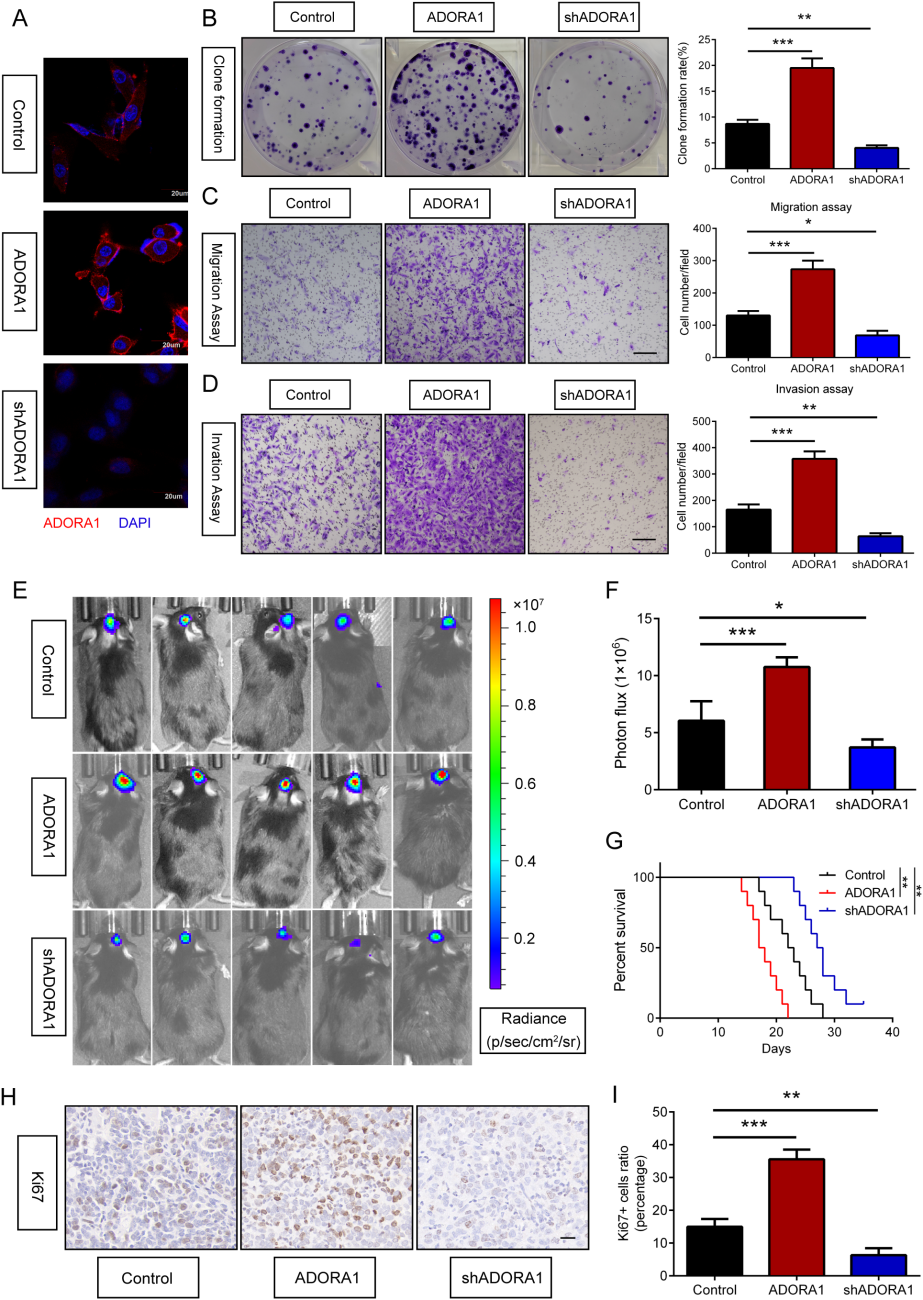
ADORA1 promotes glioma progression *in vitro* and *in vivo*. **(A)** Validation results of ADORA1 overexpression and knockdown in GBM cells. **(B)** Clone formation and quantification were performed to detect the proliferation of glioma cells transfected with LV-control, LV-ADORA1, or LV-shADORA1. **(C)** Transwell assay was performed to detect the migration capacity of glioma cells transfected with LV-control, LV-ADORA1, or LV-shADORA1, with quantification shown (scale bar: 500 μm). **(D)** Transwell assay was performed to detect the invasion capacity of glioma cells transfected with LV-control, LV-ADORA1, or LV-shADORA1, with quantification shown (scale bar: 500 μm). **(E)** Bioluminescent imaging analysis of tumor growth in xenograft C57BL/6N mice bearing GL261 glioma cells transfecting LV-control, LV-ADORA1, or LV-shADORA1 are shown. **(F)** Quantification was performed. **(G)** Survival analysis of different group mice. **(H)** Representative images of IHC staining for Ki67 in xenograft mouse brains with conditioned GL261 glioma cells (scale bar: 20 μm). **(I)** Quantification of the proportion of Ki67+ cells in xenograft mouse brains with conditioned GL261 glioma cells. *p<0.01, **p<0.001, ***p<0.001.

To explore the role of ADORA1 in glioma, we transfected GBM-Z1 glioma cells with lentivirus (LV)-control, LV-ADORA1, or LV-shADORA1 (**Figures 4A**). The results of the clone formation assay showed that *ADORA1* overexpression markedly enhanced the proliferation of glioma cells, whereas the opposite results were observed in the LV-shADORA1 group (**Figure 4B**). The results of the migration and invasion assays demonstrated that elevated ADORA1 expression significantly enhanced glioma cell motility, whereas reduced ADORA1 expression markedly diminished cell movement (**Figures 4C, D**). These findings demonstrate that ADORA1 promotes glioma progression by driving tumor cell proliferation and motility, highlighting its potential as a therapeutic target.

### 3.4 ADORA1 promotes glioma progression *in vivo*

To assess the role of ADORA1 in glioma progression *in vivo*, GL261 glioma cells transfected with LV-ADORA1 or LV-shADORA1 were implanted into mice to form orthotopic xenografts. Bioluminescent imaging revealed that mice with ADORA1-overexpressing GL261 cells exhibited stronger signals than the control group (**Figures 4E, F**). The survival analysis indicated that mice injected with GL261 cells overexpressing ADORA1 had a shorter lifespan, whereas those with GL261 cells transfected with LV-shADORA1 showed enhanced survival compared to the control group (**Figure 4G**). Ki67 staining indicated that glioma cells in mice implanted with GL261-ADORA1 showed increased proliferation compared to the control group (**Figures 4H, I**). Furthermore, GL261-implanted mice transfected with LV-shADORA1 exhibited reduced proliferative capacity (**Figures 4H, I**). These results underscore ADORA1 as a critical driver of glioma aggressiveness *in vivo*, promoting tumor growth and reducing survival.

### 3.5 ADORA1 inhibits the expression of KNG1 in glioma

**Figure 5:**
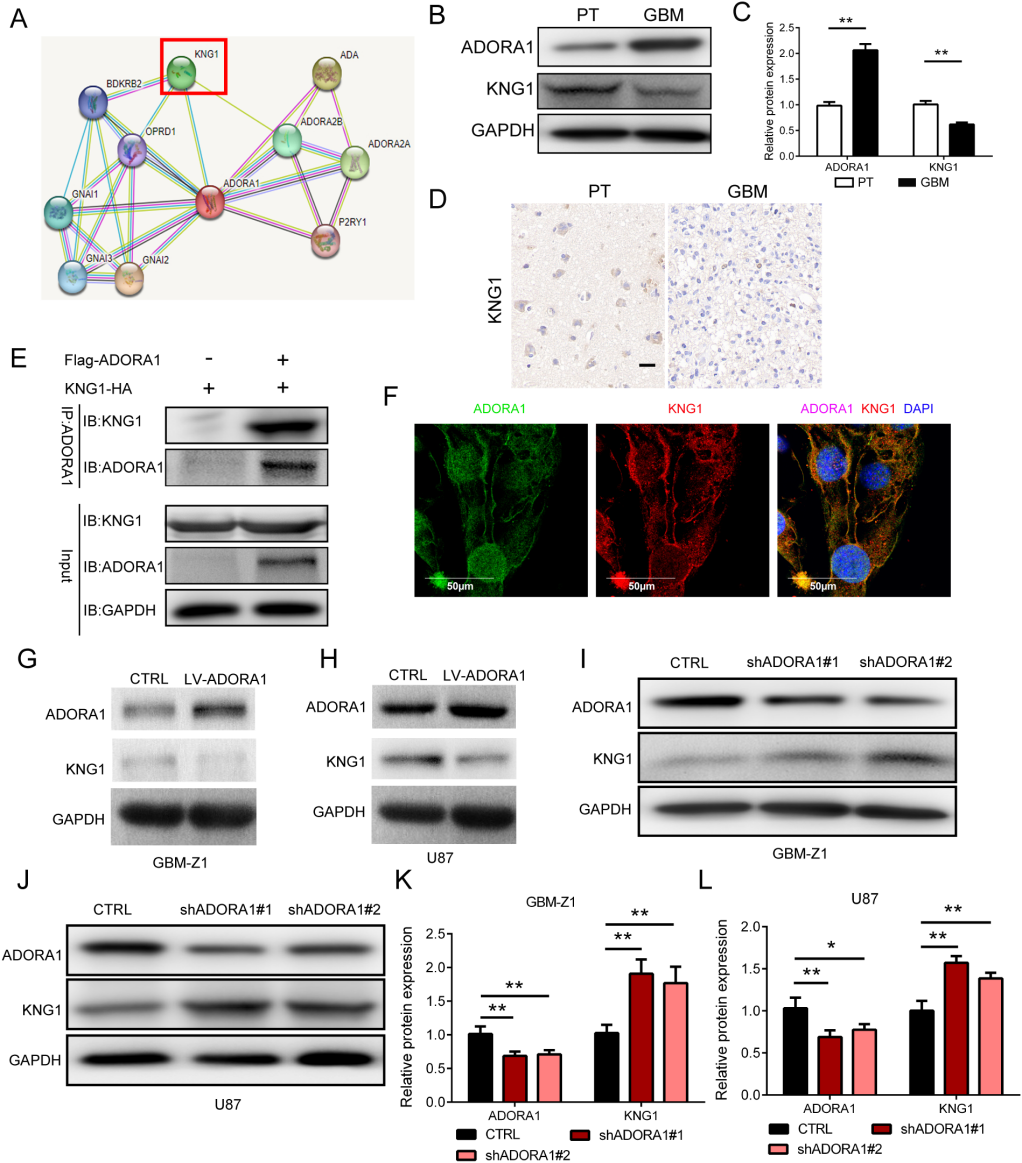
ADORA1 inhibited the protein expression of KNG1 in glioma. **(A)** Potential interactors with ADORA1 were predicted using the STRING database. **(B)** Representative images showing the protein expression levels of ADORA1 and KNG1 in human GBM and paratumor (PT). **(C)** Quantitative analysis. **(D)** Representative images of IHC staining for KNG1 in human GBM and PT (scale bars: 20 μm). **(E)** ADORA1 co-immunoprecipitates with KNG1, confirming the interaction predicted using STRING. **(F)** Immunofluorescence colocalization experiments. **(G)** Protein expression levels of ADORA1 and KNG1 in GBM-Z1 glioma cells transfecting LV-control or LV-ADORA1. **(H)** Western blot of ADORA1 and KNG1 in U87 transfecting LV-control or LV-ADORA1. **(I)** Protein expression levels of ADORA1 and KNG1 in GBM-Z1 glioma cells transfecting LV-control or LV-shADORA1. **(J)** Western blot of ADORA1 and KNG1 in U87 glioma cells transfecting LV-control or LV-shADORA1. **(J)** Protein expression levels of ADORA1 and KNG1 in U87 glioma cells. **(K)** Quantification derived from protein expression levels of ADORA1 and KNG1 in GBM-Z1 glioma cells. **(L)** Quantification derived from protein expression levels of ADORA1 and KNG1 in U87. *p<0.01, **p<0.01.

To explore the mechanism of ADORA1 in glioma progression, we searched the STRING database for potential interacting molecules. During this search, we identified KNG1, a molecule that has been linked to the progression of glioma (12), as a putative candidate (**Figure 5A**). KNG1 has the capability to penetrate tumors and undergo degradation into kinin, which in turn activates Th-1 immunity (13) and induce IFN -γ- and IL-17-producing T cells. Il-17-mediated autoimmunity and inflammation are closely linked with the administration of anti-PD-1/PD-L1 or anti-CTLA-4 antibodies in the therapeutic management of malignant neoplasms (14). Protein analysis demonstrated that higher ADORA1 protein expression level in human GBM tissue than in paratumoral tissue, but KNG1 shows the opposite result (**Figure 5B–D**). Subsequently, clinical samples were used to examine the expression of *KNG1*, revealing a noteworthy decrease in GBM tissues compared to normal tissues (**Figure 5D**). Immunoprecipitation experiments hint at an interaction between ADORA1 and KNG1 (**Figure 5E**). Co-localization experiments of ADORA1 (green staining) and KNG1 (red staining) in GBM-Z1 using confocal immunofluorescence analysis, we found that ADORA1 and KNG1 colocalized in the cell (**Figure 5F**). Western blot analysis revealed that elevated ADORA1 expression reduced KNG1 expression in GBM-Z1 and U87 glioma cells (**Figures 5G, H**). Conversely, ADORA1 knockdown resulted in increased KNG1 expression, indicating the involvement of KNG1 in this regulatory process (**Figures 5I, J, K, L**). The findings indicate that ADORA1 suppresses the expression of KNG1 in glioma, consequently impeding the anti-tumor immune response within the tumor microenvironment.

### 3.6 Overexpression of KNG1 can reverse the effect of ADORA1 on promoting glioma proliferation and angiogenesis *in vivo*

**Figure 6:**
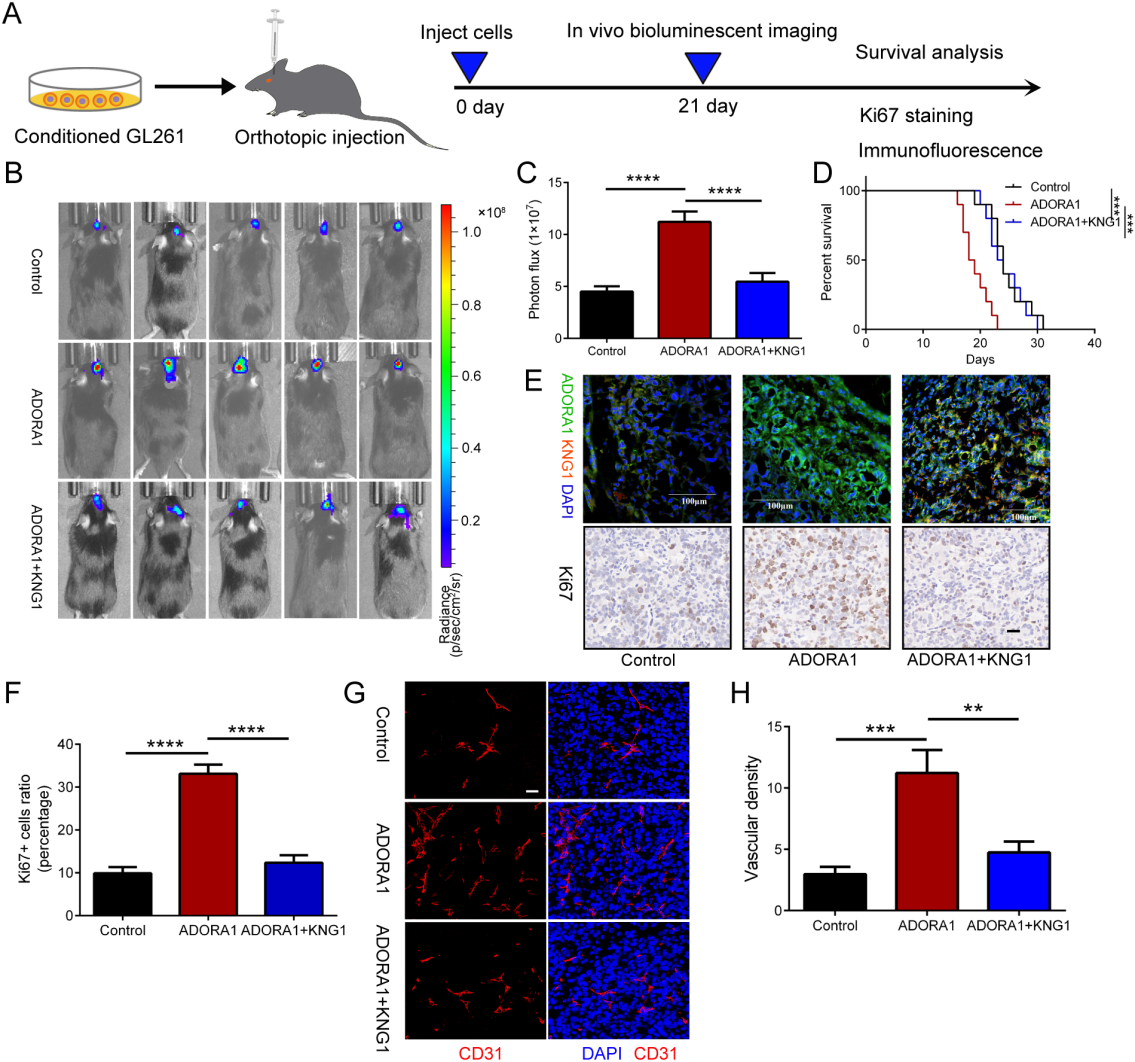
ADORA1 promotes glioma progression and angiogenesis *in vivo*. **(A)** The animal experiment flow chart. **(B)** Bioluminescent imaging analysis of tumor growth in xenograft C57BL/6N mice bearing GL261 glioma cells transfecting LV-control or LV-ADORA1 at 21 days post-implantation are shown. **(C)** Quantification was performed. **(D)** Survival analysis of different group mice. **(E)** Immunofluorescence experiments (scale bar: 100 μm) and IHC staining for Ki67 (scale bar: 20 μm) in xenograft mouse brains with conditioned GL261 glioma cells. **(F)** Quantification of the proportion of Ki67+ cells in xenograft mouse brains with conditioned GL261 glioma cells. **(G)** Immunofluorescence images of CD31 in xenograft C57BL/6N mice bearing GL261 glioma cells. Both scale bars represent 20 μm. **(H)** Quantitative analysis of relative vascular density by CD31 staining. **p<0.001, ***p<0.001, ****p<0.0001.

To investigate the impact of ADORA1 and KNG1 on glioma proliferation, we developed an *in-situ* glioma model using GL261 cells implanted in C57 mice (**Figure 6A**). On the 21st day post-implantation, we performed live bioluminescence imaging on the mice. Subsequently, we compiled survival data and collected brain tissue samples for immunohistochemical and fluorescent staining analyses. Our findings indicate that ADORA1 overexpression facilitated glioma growth and decreased survival time in the mice, whereas KNG1 overexpression mitigated these effects (**Figures 6B, C, D**). The immunohistochemical analysis revealed an increase in the proportion of ki67-positive cells in glioma tissues within the ADORA1 overexpression group, while the ki67 positivity rate diminished following KNG1 overexpression (**Figure 6E, F**). Subsequent CD31 immunofluorescence staining indicated an enhancement in vascular density in glioma tissues post-ADORA1 overexpression; however, a reduction in vascular abundance was observed when both ADORA1 and KNG1 were overexpressed concurrently (**Figure 6G, H**). These findings confirmed that the overexpression of KNG1 can counteract the proliferative effects of ADORA1 on glioma *in vivo*.

### 3.7 Inhibition of ADORA1 promotes apoptosis of glioma by KNG1

**Figure 7:**
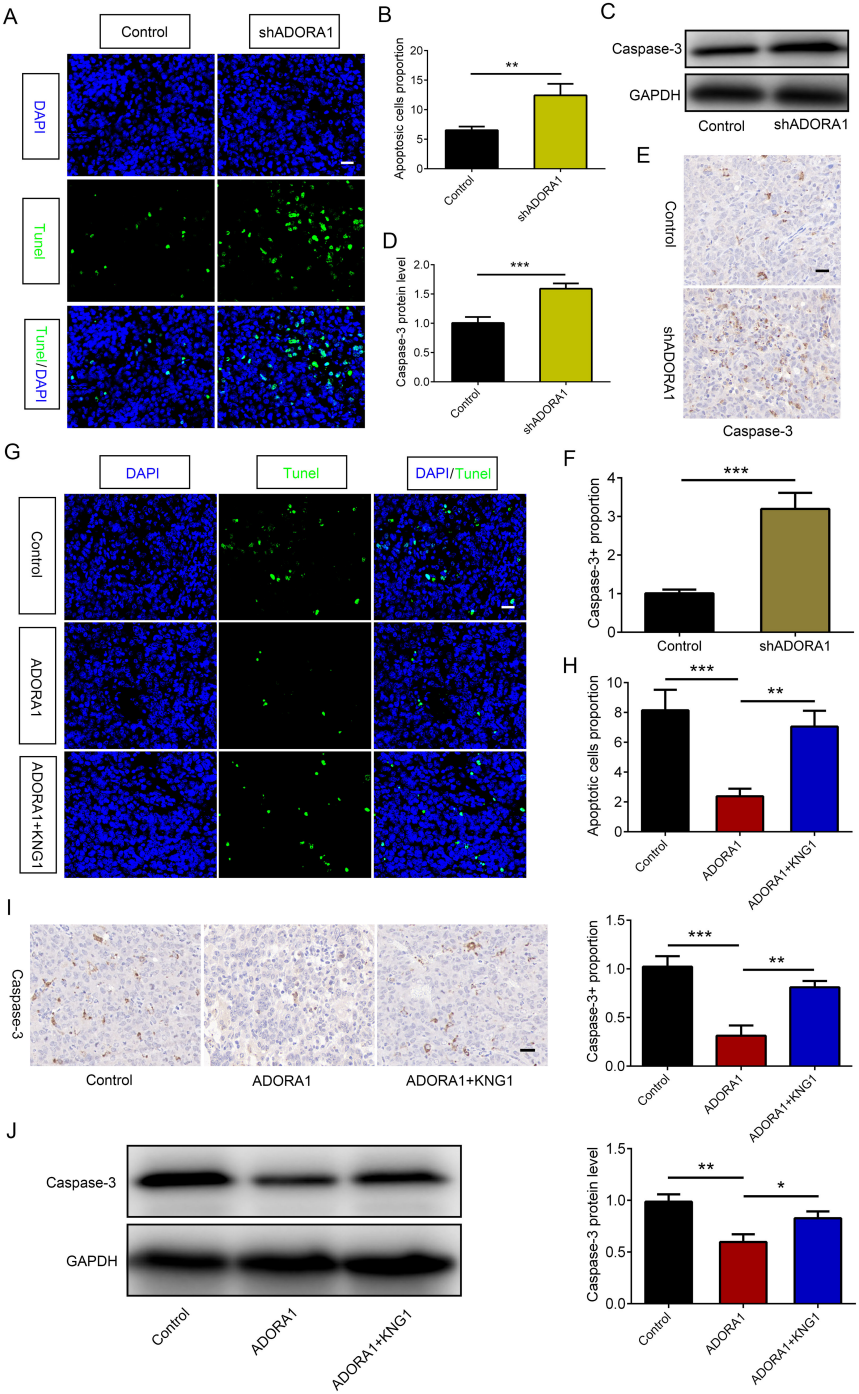
ADORA1 inhibited glioma apoptosis by KNG1. **(A, B)** Representative images of TUNEL immunofluorescence **(A)** in tumors of xenograft C57BL/6N mice bearing GL261 glioma cells and quantification **(B)** are shown in the control and shADORA1 groups (scale bar: 20 μm). **(C, D)** The protein expression level of caspase-3 **(C)** in GL261 cells and quantification **(D)** are shown in the control and shADORA1 groups. **(E, F)** IHC staing of caspase-3 **(E)** in tumors of xenograft C57BL/6N mice bearing GL261 glioma cells and quantification **(F)** are shown in the control and shADORA1 groups under different treatments. **(G, H)** Representative images of TUNEL immunofluorescence **(G)** in tumors of xenograft C57BL/6N mice bearing GL261 glioma cells and quantification **(H)** are shown under different treatments (scale bar: 20 μm). **(I J)** IHC staing and protein expression level of caspase-3 and quantification are shown in the control and shADORA1 groups. *p<0.01, **p<0.001, ***p<0.001.

To examine the role of ADORA1 in glioma cell death, GL261 glioma cells transfected with LV-shADORA1 and LV-ADORA1 were orthotopically injected into mice. The *ADORA1*-depleted group showed a significant increase in apoptosis compared to the control group, whereas the *ADORA1*-overexpressing group demonstrated a relatively lower proportion of apoptosis (**Figure 7A, B**). *In vitro*, the expression levels of caspase3 was increased in ADORA1 knockdown GL261 cells (**Figure 7C, D**). Compared with the control group, the proportion of caspase3-positive cells in ADORA1 knockdown group was increased significantly (**Figure 7E, F**). *In vivo*, we conducted additional research on the molecular mechanism of ADORA1 in suppressing glioma apoptosis. We found that KNG1 overexpression reversed the proportion of apoptosis in the *ADORA1*-overexpressing group (**Figures 7G, H**). Caspase-3 protein levels decreased in the *ADORA1* overexpression group relative to the control group (**Figures 7I, J**), while KNG1 overexpression partially reversed the reduction in caspase-3 protein in the ADORA1 overexpressing group (**Figures 7I, J**). These results suggest that ADORA1 inhibition promotes apoptosis of glioma by KNG1.

### 3.8 Inhibition of ADORA1 facilitates the enlistment of CD8+ and CD4+ T lymphocytes, amplifying responsiveness to anti-PD1 treatment in glioma

**Figure 8:**
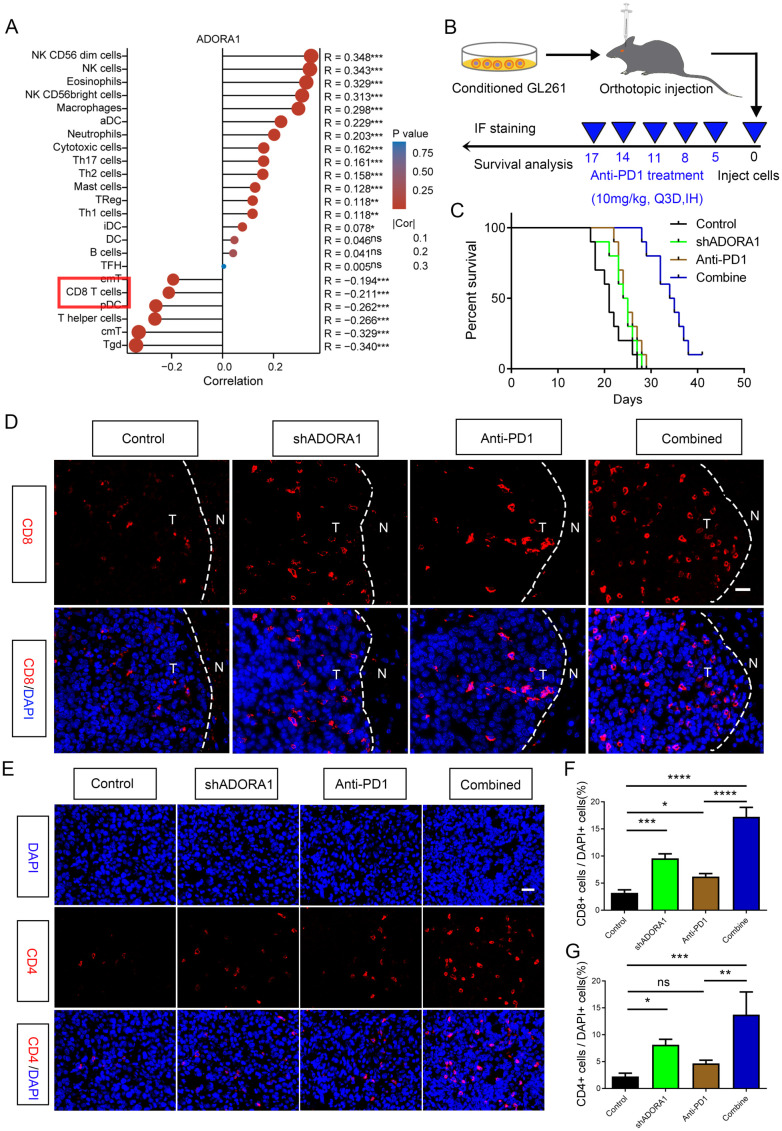
ADORA1 inhibition promoted the recruitment of CD8+ and CD4+ T cells and sensitized GBM to anti-PD1 immunotherapy. **(A)** The correlation between ADORA1 and immune cell infiltration was assessed using TCGA. **(B)** Animal experiment schematic. **(C)** Survival analysis of mice implanted with conditioned GL261 glioma cells. **(D)** Representative images of CD8 immunofluorescence in tumors of C57BL/6N xenograft mice (scale bar: 20 μm). **(E)** Immunofluorescence of CD4 in tumors of C57BL/6N xenograft mice (scale bar: 20 μm). **(F)** Quantitative analysis of the proportion of CD8+ cells in xenograft mice (n = 3). **(G)** Quantitative analysis of the proportion of CD4+ cells in xenograft mice (n = 3). *p<0.01, **p<0.001, ***p<0.001, ****p<0.0001.

Facilitating an immune-supportive tumor immune microenvironment can improve the efficacy of tumor immunotherapy. Our assy examined the effect of ADORA1 on the immune milieu of glioma. Correlation analysis using ssGSEA was performed to investigate the association between ADORA1 and various immune cell types. The results revealed a negative correlation between ADORA1 expression and CD8+ T-cell presence in glioma (**Figure 8A**). C57BL/6N mice were orthotopically injected with GL261 glioma cells transfected with shADORA1. Subsequently, we administered the PD1 mAb to evaluate its tumor-fighting potential (**Figure 8B**). The combination of shADORA1 and anti-PD1 therapy improved survival compared to the control group (**Figure 8C**). Moreover, the anti-PD1 combined with shADORA1 group had a longer survival time compared to the anti-PD1 group (**Figure 8C**). Inhibiting ADORA1 increased the numbers of CD8+ and CD4+ T cells (**Figures 8D–G**). The combined treatment group exhibited the greatest abundance of CD8+ and CD4+ T cells, significantly surpassing the levels observed in the anti-PD1 treatment group (**Figures 8D–G**). The results indicated that inhibiting ADORA1 increased the recruitment of CD8+ and CD4+ T cells in gliomas, thereby benefiting from PD-L1-targeted immunotherapy.

## 4 Discussion

Patients diagnosed with GBM experience adverse outcomes, with a typical survival period of about 15 months, despite undergoing aggressive treatment as commonly recommended (15). Bioinformatics analysis revealed that GBM exhibited elevated *ADORA1* expression, which was positively correlated with malignancy-associated clinical features such as IDH wild-type and 1p/19q non-co-deletion, as indicated by public databases (16). ADORA1 was identified as a marker of unfavorable prognosis according to AUC and survival analyses. In addition, we confirmed that the expression level of ADORA1 in gliomas was significantly up-regulated by western blotting and IHC staining. These findings offer insight into the potential of ADORA1 as a predictive marker of glioma.

ADORA1 has pro-cancer effects against cancer progression (7–9). The influence of ADORA on tumor proliferation and migration encompasses the cross-regulation of various pathways, including classical signal transduction pathways such as PI3K/AKT (17), and metabolic reprogramming (9, 18). Previous studies have reported that ADORA1 was highly expressed in high-grade gliomas (18), however, the effect of ADORA1 on glioma progression and glioma immunity therapy was unclear. According to our research, excessive ADORA1 expression significantly facilitated the growth, movement, and infiltration of glioma cells, which are associated with the progression of glioma. We screened molecules that potentially interact with ADORA1 using the STRING database and identified KNG1 as a putative candidate. We further demonstrated that ADORA1 induces glioma progression by inhibiting KNG1. KNG1, a precursor of kinins in the kallikrein-kinin system, induces apoptosis and exerts anti-cancer effects on glioma (12). Malignant gliomas are characterized by an inherent resistance to apoptosis, a regulated form of programmed cell death (19, 20). Our study further demonstrated that ADORA1 inhibition promoted glioma apoptosis induced by KNG1.

Immunotherapy, which relies on boosting the capacity of immune cells to eliminate cancer cells, has revolutionized cancer treatment. Data from various omics studies and clinical samples highlight the increased expression of PD-L1 in GBM, suggesting its potential as a promising target for immunotherapy (21, 22). Preclinical findings indicate that anti-PD-1/PD-L1 therapy can induce a shift in macrophage polarization from the M2 to the M1 phenotype, thereby converting the immunosuppressive microenvironment into a pro-inflammatory state, ultimately leading to prolonged survival in mice afflicted with GBM (23). Furthermore, preclinical models demonstrate that early inhibition of LAG3 significantly enhances prognosis in GBM-bearing mice and is highly effective in tumor eradication when combined with anti-PD-1/PD-L1 therapy (24). Despite these promising preclinical outcomes, clinical trials involving PD-1, CTLA-4, and other immunotherapies for GBM have unfortunately not achieved substantial success (2, 25). KNG1 enhances Th-1 immune responses and stimulates the production of IFN-γ and IL-17 by T cells. IL-17-mediated autoimmunity and inflammation is closely linked to the administration of anti-PD-1/PD-L1 or anti-CTLA-4 antibodies (13, 26, 27). ADORA1 suppresses the expression of KNG1 in glioma, consequently impeding the anti-tumor immune response within the tumor microenvironment. We demonstrated the effect of ADORA1 on T cells, showing that inhibition of ADORA1 in the shADORA1 group increased the recruitment of CD4+ T and CD8+ T cells to gliomas compared to the control group. During the immune response against tumors, CD8+ T cells secrete cytotoxic substances to specifically identify and eliminate cancerous cells. The inhibition of ADORA1 led to the stimulation of CD8+ T cells, thereby boosting the immune response to tumors.

Patients with cancer have benefited from PD-1/PD-L1 mAb immune checkpoint blockade (28). However, the overall response rate to PD-1 or PD-L1 monoclonal antibody therapy rarely exceeds 40% (29). Hence, the emergence of different treatment approaches, such as the use of combined therapies, has garnered significant attention in the field of cancer treatment. Our results showed that combining ADORA1 blockade with PD-1 monoclonal antibody treatment significantly improved T-cell recruitment. The inhibition of ADORA1 greatly enhanced the effectiveness of PD1 monoclonal antibodies in treating glioma, resulting in a notable decrease in tumor size and an extended period of survival. This indicates that ADORA1 inhibition increased the responsiveness of glioma to anti-PD1 treatment. Moreover, evidence from studies on melanoma and lung cancer demonstrates that ADORA1 deficiency combined with PD-1 mAb treatment supports our perspective (30). Therefore, the use of combined therapeutic approaches may represent a novel strategy to enhance the therapeutic outcomes of individuals with glioma.

## 5 Conclusion

Our results indicated that ADORA1 was highly expressed in gliomas and that ADORA1 overexpression promoted glioma progression by inhibiting KNG1. Inhibiting ADORA1 promoted glioma cell death via KNG1, increased T-cell recruitment, and improved glioma responsiveness to anti-PD1 therapy. Therefore, ADORA1 inhibition might exert therapeutic effects against glioma.

## Data Availability

Publicly available datasets were analyzed in this study. This data can be found here: The Cancer Genome Atlas (TCGA) (https://www.cancer.gov/), Chinese Glioma Genome Atlas (CGGA) (http://www.cgga.org.cn/), and Genotype-Tissue Expression (GTEx) databases (https://www.gtexportal.org/).
